# Selective Hydrogenation of Formamide to Methanol Over Supported Platinum Catalysts

**DOI:** 10.1002/anie.202522476

**Published:** 2026-05-08

**Authors:** Xuetao Qin, XinXin Tian, Shou Qiu, Han Yan, Haijun Jiao, Henrik Junge, Matthias Beller

**Affiliations:** ^1^ Leibniz‐Institut Für Katalyse e.V. Rostock Germany; ^2^ Institute of Molecular Science Shanxi University Taiyuan People's Republic of China; ^3^ Beijing National Laboratory For Molecular Engineering New Cornerstone Science Laboratory College of Chemistry and Molecular Engineering Peking University Beijing People's Republic of China; ^4^ Hefei National Research Center for Physical Sciences at the Microscale Key Laboratory of Strongly‐Coupled Quantum Matter Physics of Chinese Academy of Sciences Key Laboratory of Surface and Interface Chemistry and Energy Catalysis of Anhui Higher Education Institutes Department of Chemical Physics University of Science and Technology of China Hefei People's Republic of China

**Keywords:** CO_2_ utilization, methanol synthesis, platinum catalysts, selective hydrogenation, strong metal–support interaction

## Abstract

The rising global energy demand, driven by population and economic growth, continues to be met largely by fossil fuels. Consequently, atmospheric CO_2_ concentration has increased from ∼280 ppm in the pre‐industrial era to over 430 ppm today, motivating integrated capture‐and‐conversion strategies that valorize CO_2_ as a feedstock. Amines are widely employed for post‐combustion CO_2_ capture due to their low cost, rapid kinetics, and reversible carbamate formation. These amine–CO_2_ adducts can be hydrogenated to formamides and subsequently to methanol under mild conditions (<150°C), regenerating the amine. However, selective C─N bond cleavage during the formamide hydrogenation for efficient release of methanol without sorbent deactivation remains a key challenge. Here, we demonstrate that 1 wt% Pt supported on TiO_2_ (commercial P25) catalyzes the conversion of 4‐formylmorpholine to methanol with up to 62% yield and 95% selectivity at 150°C. In situ characterizations and DFT computations reveal that strong interaction between dispersed Pt and the support promotes selective C─N hydrogenolysis. The catalyst also exhibits excellent stability. These findings establish a robust heterogeneous platform for combined CO_2_ capture and methanol synthesis, highlighting the critical role of support‐induced electronic effects in controlling bond scission.

## Graphical abstract

Amine–CO_2_ adducts can be hydrogenated to formamides and subsequently to methanol under mild conditions (<150°C), establishing an integrated CO_2_ capture‐and‐conversion route in a heterogeneous system that directly links amine sorbent cycling with methanol synthesis.

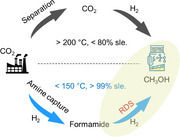

## 1 Introduction

Global energy demand continues to increase, driven by population growth, economic development, and the pursuit of higher standards of living. Despite the rapid expansion of renewable energy technologies, this demand remains met predominantly by fossil‐fuel combustion. As a consequence, the atmospheric concentration of CO_2_, the principal anthropogenic greenhouse gas, has risen from its pre‐industrial baseline of ∼280 to ∼430 ppm today [1]. This unprecedented increase has accelerated global warming and climate change, prompting urgent international efforts to mitigate emissions, stabilize atmospheric CO_2_ levels, and transition toward a sustainable energy economy. Central to these efforts is the development of technologies that not only capture CO_2_ from concentrated industrial sources and dilute ambient air but also valorize it as a feedstock for fuels and materials [2, 3, 4]. Such strategies address both climate and resource challenges, creating an economic and technological pathway toward a CO_2_‐neutral, and ultimately CO_2_‐negative, global economy [5, 6, 7].

**FIGURE 1:**
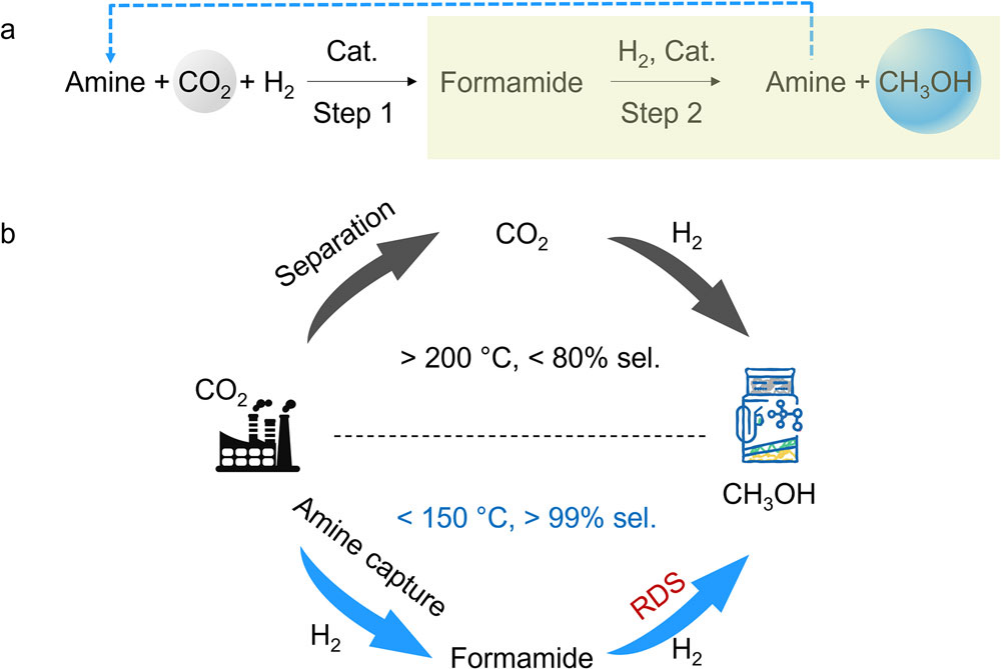
Illustration of CO_2_ hydrogenation to methanol. (a) Catalytic CO_2_ hydrogenation to methanol in the presence of amine. Cat., catalyst. (b) Comparison of CO_2_ hydrogenation to the methanol reaction pathways described in this work. Blue arrows represent CO_2_ hydrogenation to methanol in the presence of amine, while black ones show conventional reaction pathways including separation and CO_2_ hydrogenation. RDS, rate‐determining step. sel., selectivity. The reaction shaded in pale yellow represents the central focus of this study.

Among the various CO_2_ capture technologies, amine‐based sorbents remain the benchmark for post‐combustion applications due to their low cost, rapid absorption kinetics, chemical tunability, and compatibility with continuous‐flow operation [8]. The reversible formation of carbamate adducts provides both a practical means of capturing CO_2_ and a chemical handle for subsequent conversion. Importantly, these adducts lower the thermodynamic and kinetic barriers for downstream transformations, offering opportunities to couple CO_2_ capture directly with its conversion into value‐added chemicals under relatively mild conditions [9, 10, 11]. Such integrated capture‐and‐conversion approaches not only reduce process complexity but also improve the overall economics and carbon efficiency of CO_2_ utilization technologies. Significant progress has been made over the past decade in catalytic pathways for CO_2_ hydrogenation. One particularly promising strategy involves the hydrogenation of CO_2_ in the presence of amines to form formamides (Figure 1a, Step 1). Both homogeneous catalysts [12, 13, 14] and heterogeneous systems [15, 16] have been developed, achieving improved activities, higher selectivities, and enhanced recyclability under increasingly mild conditions. These advances demonstrate that formamides can serve as practical intermediates for the chemical upgrading of captured CO_2_. However, to fully close the carbon cycle, further hydrogenation of formamides is required. This second step not only produces methanol, a liquid fuel and versatile platform molecule central to the methanol economy, but also regenerates the amine sorbent for repeated CO_2_ capture (Figure 1a, Step 2). Together, these two steps establish a closed‐loop capture‐and‐conversion cycle with considerable commercial and environmental potential [17, 18]. Despite this promise, significant challenges remain. The selective hydrogenation of formamides to methanol is hindered by competing reaction pathways, particularly C─O bond cleavage, which generates *N*‐methylated amines instead of releasing methanol. This undesired pathway compromises both product selectivity and amine recyclability, undermining the integration of capture and conversion. Overcoming these limitations requires catalysts capable of selectively activating and cleaving C─N bonds under mild conditions, while maintaining stability and reusability. Achieving this goal would establish a robust platform for coupling CO_2_ capture with methanol synthesis in a single, sustainable cycle.

Unlike conventional gas‐phase CO_2_ hydrogenation to methanol [19, 20, 21], which requires high temperatures and affords only moderate selectivity, the amine‐promoted route proceeds under milder conditions (<150°C) while delivering methanol with near‐quantitative selectivity (Figure 1b). In the first step, CO_2_ is efficiently captured by morpholine solution and converted to 4‐formylmorpholine via catalytic formylation (Figure 1a, Step 1). A highly active heterogeneous iridium catalyst has recently been reported, achieving excellent productivity (catalyst turnover number of >5,000,000 in a single batch) and >99% selectivity to 4‐formylmorpholine [16]. These advances indicate that formamide generation is not a limiting factor in the overall reaction sequence. The true bottleneck lies in the subsequent selective hydrogenation of formamides to methanol (Figure 1a, Step 2) [22]. Undesired C─O bond cleavage leads to *N*‐methylated amines, irreversibly converting the secondary amine sorbent into a tertiary amine and thereby halting further CO_2_ capture. In contrast, selective C─N bond cleavage yields methanol while simultaneously regenerating the “active” amine, enabling continued uptake (Figure 1a). To date, most catalysts capable of achieving such selectivity have been homogeneous systems. Milstein and coworkers as well as our group, for example, have demonstrated Ru–PNN and Mn–PNN pincer complexes that catalyze amide hydrogenation to alcohols via C─N bond scission [23, 24, 25]. Similarly, the groups of Prakash and Beller reported amine‐assisted homogeneous Ru catalysts for direct CO_2_ hydrogenation to methanol [26, 27]. While highly effective, homogeneous catalysts face challenges in separation, continuous operation, and tolerance to the trace impurities typical of flue‐gas streams. By contrast, heterogeneous catalysts are better suited for industrial implementation due to their stability, ease of recycling, and integration into flow processes. Yet, despite their advantages, heterogeneous approaches for CO_2_ hydrogenation in the presence of amines, particularly selective hydrogenation of formamides to methanol, remain only sparsely explored. Although in previous studies the viability of the formamide intermediate pathway from CO_2_ and H_2_ to methanol was established [28, 29, 30], and some heterogeneous catalysts were developed, the challenge selectively hydrogenating formamides has limited the progress toward practical, integrated capture‐and‐conversion concepts.

Here, we address this challenge by focusing on the second step: selective hydrogenation of formamides to methanol while regenerating the CO_2_ sorbent (Figure 1b). Using 4‐formylmorpholine (also known as *N*‐formylmorpholine, NFM) as a model substrate, we probe the competition between C─O and C─N bond scission. We demonstrate that a 1 wt% Pt/TiO_2_ (P25) catalyst achieves up to 95% selectivity for C─N cleavage at 150°C. In situ characterizations reveal that unusually electron‐rich Pt sites, generated through strong metal–support interactions (SMSI) with TiO_2_ [31, 32], are crucial to this performance. These findings not only establish a heterogeneous pathway for amine‐promoted CO_2_‐to‐methanol conversion but also provide broader insights into the selective hydrogenation of amide groups, which are central to the synthesis of pharmaceuticals, natural products, and agrochemicals. The Pt/P25 heterogeneous catalyst, achieving highly selective C─N bond cleavage in formamide hydrogenation at such mild conditions, offers new potential for an integrated CO_2_ capture‐and‐conversion cycle.

## 2 Results

We selected NFM as our model substrate because it is commercially available, high‐boiling, and operationally robust; it affords a clean and easily quantifiable hydrogenolysis product profile; it is a recognized benchmark in formamide hydrogenation studies; and it has direct relevance to emerging reversible CO_2_‐utilization cycles [33, 34]. Conventional formamide hydrogenation typically requires temperatures above 150°C, elev ated hydrogen pressures, and base additives [35, 36]. Here, we aimed to achieve selective hydrogenation of NFM at 150°C under comparatively mild conditions (50 bar H_2_).

We first evaluated a series of noble‐metal catalysts (Pt, Ru, Rh, Ir, Pd; 1 wt% loading) supported on carbon black (Table 1, entries 2–6). A blank experiment without a catalyst confirmed that the autoclave itself is catalytically inert (Table 1, entry 1). Among the metal/C catalysts, Ru and Pd (Table 1, entries 2 and 4) showed appreciable activity but predominantly yielded the undesired C─O cleavage product 4‐methylmorpholine (also known as *N*‐methylmorpholine, NMM), which irreversibly deactivates the amine and prevents its reuse for CO_2_ capture [17, 37, 38, 39]. This is in line with previous studies of formamide hydrogenolysis, which commonly observe that Pd and Ru systems preferentially break the C─O bond [40]. Rh and Ir (Table 1, entries 3 and 5) displayed only moderate activity and selectivity. By contrast, Pt/C (Table 1, entry 6) delivered the highest conversion along with 81.6% selectivity to methanol and 86.5% selectivity to morpholine. Accordingly, Pt was selected for further studies.

**TABLE 1:** Selective hydrogenation of 4‐formylmorpholine over various heterogeneous catalysts.

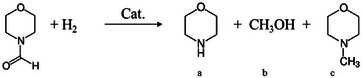					
Entry	Catalysts	Conversion/%	Selectivity (%)		
			a	b	c
1	Blank	n.d.	—	—	—
2	1%Ru/C	30.2	n.d.	n.d.	100.0
3	1%Rh/C	18.6	56.2	53.0	43.8
4	1%Pd/C	18.5	6.5	3.6	93.5
5	1%Ir/C	9.6	42.6	40.1	57.4
6	1%Pt/C	44.7	86.5	81.6	13.5
7	1%Pt/Al_2_O_3_	45.5	65.8	60.7	34.2
8	1%Pt/MgO	52.8	56.2	51.8	43.8
9	1%Pt/H‐ZSM‐5	32.5	100.0	94.8	n.d.
10	1%Pt/CeO_2_	50.1	92.3	88.6	7.7
11	1%Pt/P25	62.3	100.0	95.2	n.d.
12	0.1%Pt/P25	10.6	100.0	94.6	n.d.
13	0.5%Pt/P25	28.4	100.0	95.3	n.d.
14	2%Pt/P25	70.8	89.8	85.6	10.2

General reaction conditions: 100 mg catalyst, 20 mmol 4‐formylmorpholine, 50 bar H_2_, 30 mL 1,4‐dioxane, 150°C, 12 h.

To probe the influence of supports, we loaded 1 wt% Pt onto a range of materials: inert supports (C, Al_2_O_3_), redox‐active oxides (CeO_2_, TiO_2_–P25), an acidic oxide (H‐ZSM‐5), and a basic oxide (MgO) (Table 1, entries 6–11). Pt/Al_2_O_3_ (Table 1, entry 7) gave conversion comparable to Pt/C but lower methanol selectivity. Pt/MgO (Table 1, entry 8) increased conversion but at the expense of selectivity. Pt/H‐ZSM‐5 (Table 1, entry 9) afforded high methanol (94.8%) and morpholine (∼100%) selectivities, but its NFM conversion was lower, likely due to strong acid–base interactions between morpholine and the zeolite framework [41, 42]. In contrast, the redox‐active supports CeO_2_ and P25 (Table 1, entries 10 and 11) delivered both high conversion and high methanol selectivity. Notably, Pt/P25 achieved 62.3% conversion with 95.2% methanol selectivity, making it the most promising system, obtaining among the highest methanol yields under mild, additive‐free conditions compared to the best state‐of‐the‐art homogeneous and heterogeneous catalysts (Table S1). We also examined the effects of solvent and reaction temperature. Screening of solvents revealed that 1,4‐dioxane provided the best balance of conversion and methanol selectivity (Table S2). The reaction temperature strongly influenced the activity: at 120°C, NFM conversion was only ∼10%, while at 150°C conversion exceeded 62% with methanol selectivity >95% and morpholine selectivity ∼100% (Table S3). The latter conditions enable complete recycling of the amine for CO_2_ capture.

Finally, to investigate the role of Pt loading, we synthesized Pt/P25 catalysts with different Pt contents. NFM conversion increased with Pt loading, from 10.6% for 0.1 wt% Pt to 70.8% for 2 wt% Pt (Table 1, entries 11–14). However, selectivity decreased above 1 wt%: while catalysts with ≤1 wt% Pt maintained ∼95% methanol selectivity (Table 1, entries 11–13), the 2 wt% sample dropped to 85.6% (Table 1, entry 14). These trends likely reflect changes in Pt dispersion and electronic structure with loading, consistent with strong metal–support interactions (SMSI) between Pt and P25. Detailed mechanistic investigations of these effects are presented in the following sections.

**FIGURE 2:**
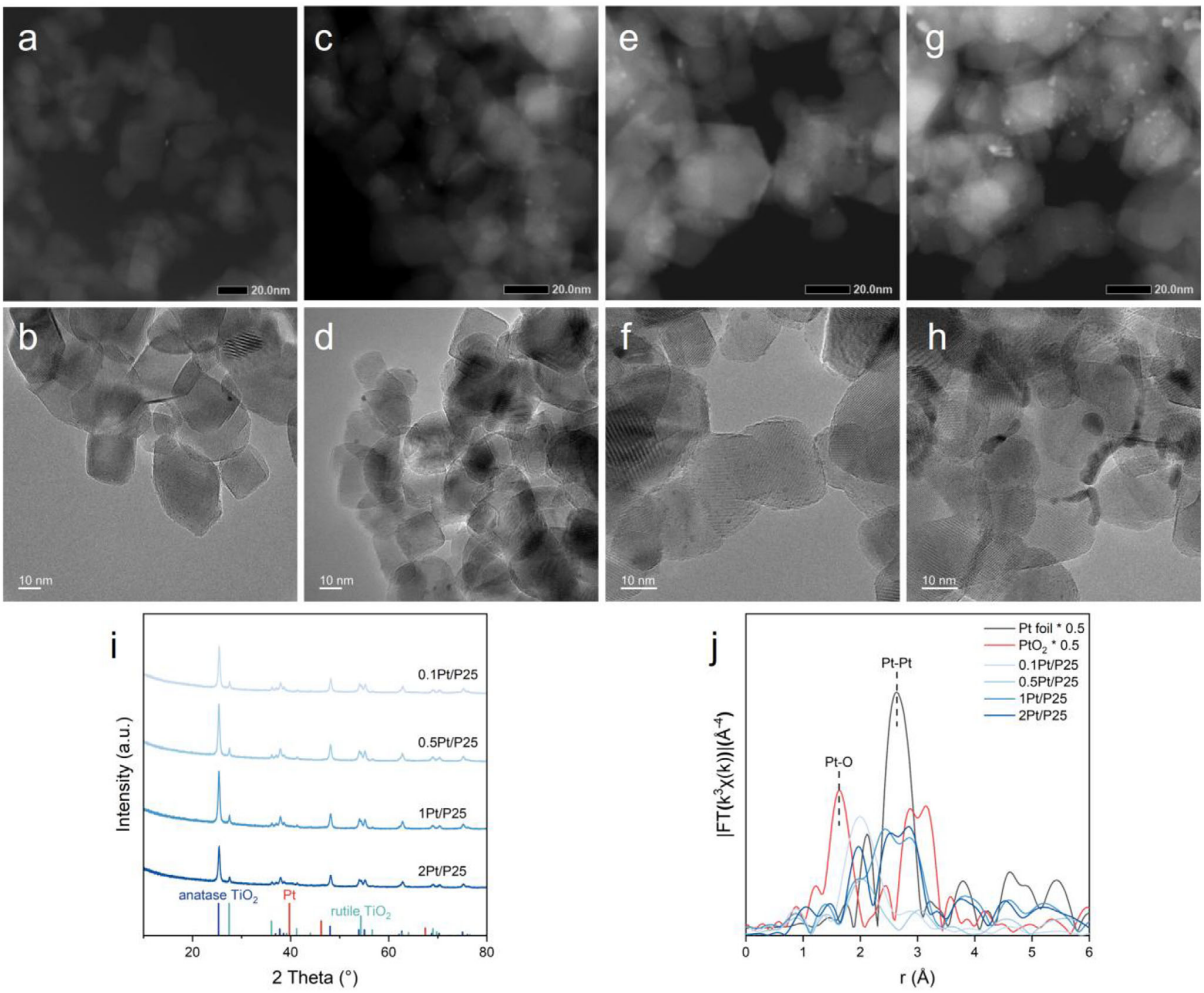
Characterization of different Pt/P25 catalysts. (a, b) High‐angle annular dark‐field scanning transmission electron microscopy (HAADF‐STEM) and high‐resolution transmission electron microscopy (HRTEM) images of 0.1%Pt/P25. (c, d) HAADF‐STEM (c) and HRTEM (d) images of 0.5%Pt/P25. (e, f) HAADF‐STEM (e) and HRTEM (f) images of 1%Pt/P25. (g, h) HAADF‐STEM (g) and HRTEM (h) images of 2%Pt/P25. (i) XRD profiles of different Pt/P25 catalysts. (j) Pt *L_3_*‐edge EXAFS for different Pt/P25 catalysts. The data are *k* ^3^‐weighted and without phase‐corrected.

To elucidate the atomic‐scale structure of the Pt/P25 catalysts, high‐angle annular dark‐field scanning transmission electron microscopy (HAADF‐STEM), high‐resolution transmission electron microscopy (HRTEM), and x‐ray absorption spectroscopy (XAS) were performed. HAADF‐STEM and HRTEM images of the 0.1%Pt/P25 sample showed that Pt is highly dispersed on P25, with only a few isolated clusters of 1‐3 nm (Figures 2a,b, S7, and S8). In the 0.5% (Figures 2c,d, S9, and S10) and 1%Pt/P25 (Figures 2e,f, S11, and S12) samples, the cluster size is essentially unchanged, but their density increases with higher loading. In contrast, HAADF‐STEM and HRTEM images of the 2%Pt/P25 catalyst reveal the appearance of Pt nanoparticles (Figures 2g,h, S13, and S14). The absence of discernible Pt diffraction peaks (Figure 2i) is consistent with the low Pt loading and the presence of predominantly small Pt species, rather than being conclusive evidence of uniform dispersion alone. While HAADF‐STEM images (Figures 2g and S13) reveal the presence of some Pt nanoparticles, the absence of XRD features suggests that extensive growth of large crystalline Pt particles did not occur due to the low Pt loading.

Extended X‐ray absorption fine‐structure (EXAFS) analysis corroborates the microscopy evidence. The Pt‐O and Pt‐Pt contributions in the Pt/P25 series exhibit intermediate amplitudes relative to PtO_2_ and Pt foil (Figure 2j), indicating that Pt resides as small metal clusters rather than as large particles or isolated cations. Wavelet‐transform of the Pt *L*_3_‐edge (Figures S15–S20) place the first‐shell Pt‐O feature of PtO_2_ at (*k, R*) = (5.5, 1.8) and the Pt‐Pt feature of Pt foil at (11.2, 2.8) (Figures S15 and S16) [43, 44]. The maxima for all Pt/P25 samples lie between these two loci, confirming the simultaneous presence of Pt‐O and Pt‐Pt coordination (Figures S17–S20). EXAFS curve fitting (Figure S21) results yield a Pt‐O distance of ∼2.0 Å with a coordination number that falls from 4 to 1, alongside a Pt‐Pt distance of ∼2.7 Å with a coordination number that rises from 4 to 7 as the Pt loading increases (Table S4). This is fully consistent with the XRD and HAADF‐STEM observations. Taken together, these results demonstrate that in the Pt/P25 catalysts, Pt is dispersed as clusters on the TiO2 support.

**FIGURE 3:**
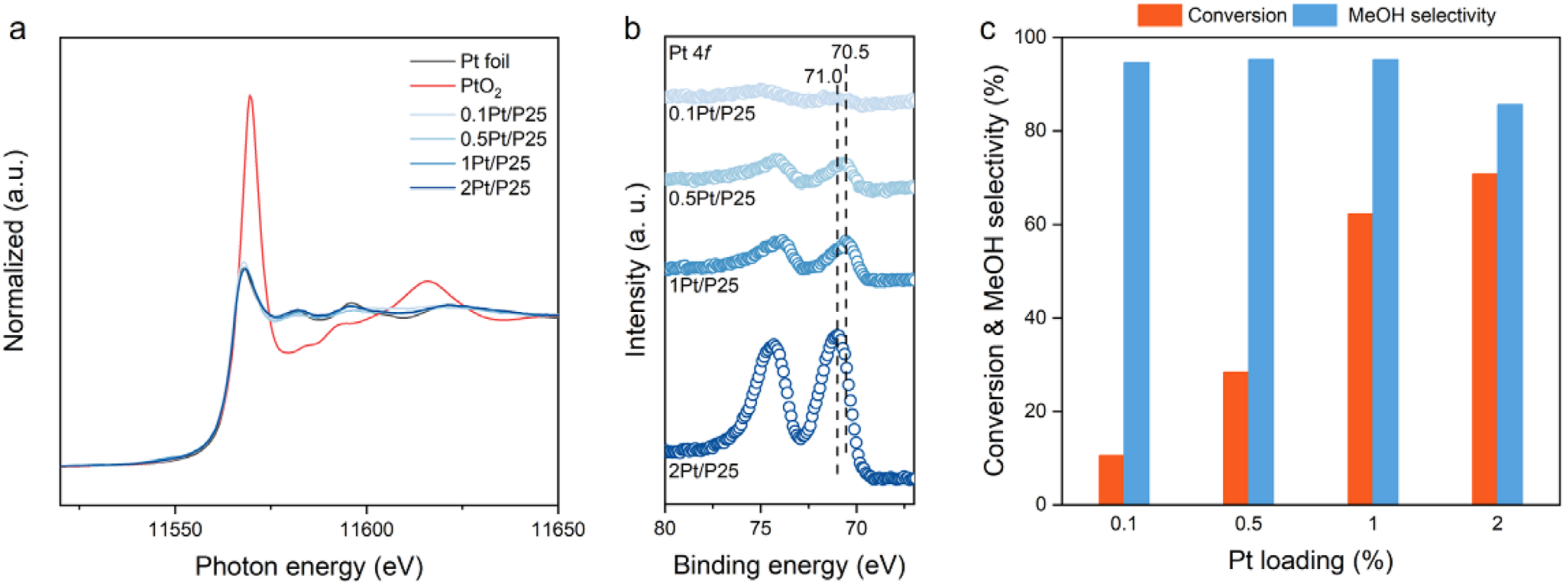
Characterization and reaction performance of Pt/P25 catalysts with different Pt states. (a) In situ Pt *L_3_*‐edge XANES of various Pt/P25 catalysts. (b) In situ XPS of Pt 4*f* spectrum for various Pt/P25 catalysts. (c) Conversion and methanol selectivity as a function of Pt loading.

Quasi in situ x‐ray absorption spectroscopy (XAS) and in situ x‐ray photoelectron spectroscopy (XPS), performed by transferring pre‐treated samples directly into the measurement chamber without air exposure, were used to probe the electronic structure of Pt/P25 catalysts. Pt *L*_3_‐edge X‐ray absorption near‐edge structure (XANES) spectra confirm that, after reduction, Pt species in all Pt/P25 catalysts are predominantly metallic (Figure 3a). In the 0.1%Pt/P25 sample, however, the Pt valence state is slightly higher than that of metallic Pt, consistent with very small clusters strongly influenced by the oxide support. This observation aligns with HAADF‐STEM results, indicating that Pt exists as highly dispersed clusters at low loading. Quasi in situ XPS provides further insight into the near‐surface electronic states (Figures 3b and S22). The spectra are dominated by features at 71.0 (Pt^0^) and 70.5 eV, assigned to electron‐rich Pt^δ–^ species [45, 46]. The latter arises from electron transfer from TiO_2_ to Pt, characteristic of strong metal–support interactions (SMSI) in Pt/TiO_2_ systems. At 2%Pt/P25, Pt is present mainly as metallic Pt^0^, in agreement with HAADF‐STEM images that reveal nanoparticle formation. In contrast, at 0.5% and 1%Pt/P25 (the 0.1% sample provided insufficient signal for detailed analysis), the 70.5 eV peak predominates, demonstrating that reduced catalysts generate a large fraction of electron‐rich Pt^δ–^ species. This electronic enrichment likely underpins their high methanol selectivity in NFM hydrogenation.

To understand how such a slight change in Pt oxidation states drastically changes the catalytic selectivity, we investigated NFM hydrogenation using DFT methods. The simplified Gibbs free energy profiles are shown in Figure 4. Computational details are provided in the Supporting Information (Figures S25–S37).

**FIGURE 4:**
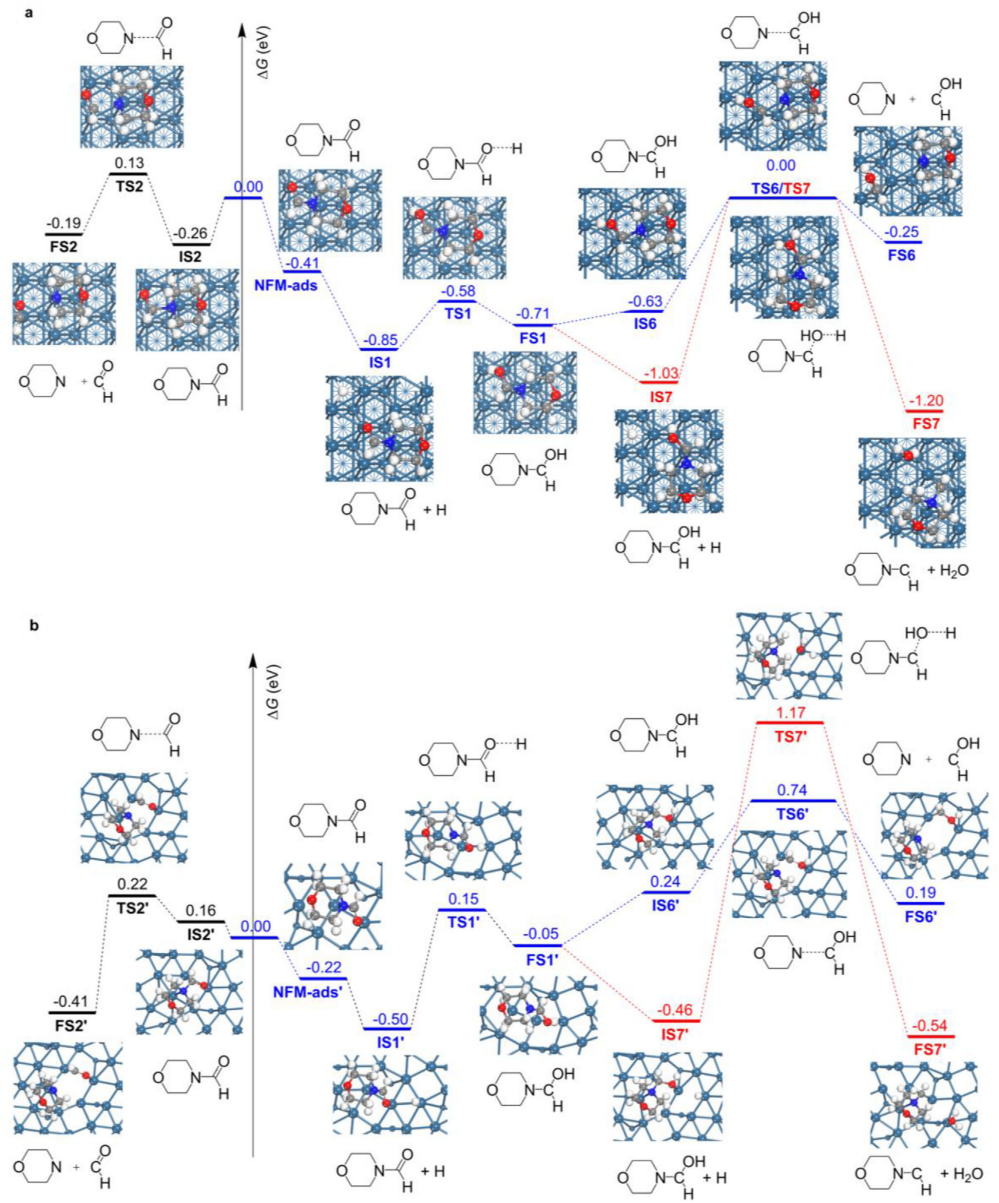
Potential energy diagram of NFM possible reaction pathways on (a) Pt(111) and (b) Pt/TiO_2_(101) surfaces. White, gray, blue, red, and cyan spheres represent H, C, N, O, and Pt atoms, respectively.

To mimic large Pt metal clusters, we computed the reaction on the metallic Pt(111) surface and found that NFM tends to interact preferably with the surface via the formyl O atom (Figure S26, Table S5). Starting from the adsorbed NFM, we computed four different reaction paths (Figures S27 and S29) and identified the most favored one (Figure 4a), having the surface hydrogen addition to the adsorbed formyl O atom resulting in the formation of NHMM (4‐(hydroxymethylene)morpholin‐4‐ium) with a Gibbs free energy barrier of 0.27 eV (**TS1**). Next, there are two reaction paths for the NHMM (Figures S30–S33). One undergoes the direct C─N bond cleavage (**TS6**) resulting in the formation of hydroxyl methylene (CHOH) and morpholin‐4‐ide (**FS6**), which can be hydrogenated to methanol and morpholine. The second path undergoes the hydrogenative C‐O cleavage (**TS7**), resulting in the formation of H_2_O and morpholin‐4‐ium‐4‐ylidenemethanide (**FS7**), which can be further hydrogenated to NMM as the side product. As shown in Figure 4a, both reactions have the same apparent Gibbs free energy barriers, although the formation of NMM is thermodynamically more favored. In addition, the direct C─N bond dissociation of NFM has a higher apparent barrier (**TS2**) than **TS6/TS7** by 0.13 eV, ruling out the direct C‐N bond cleavage process. This indicates that on large metallic Pt clusters, this hydrogenation reaction should not be selective.

On the Pt/TiO_2_(101) surface, the most stable adsorption configuration has the formyl O atom of NFM on the positively charged Pt atom (Pt1, Figure S35, −0.22 eV), and that of NFM interacting via C, N, and O atoms with the negatively charged Pt atoms (Pt2 and Pt3) is endothermic (Figure S35, 0.16 eV). Starting from the most stable adsorption configuration, we focused our study on these four key steps on the Pt/TiO_2_(101) surface (Figures S36 and S37). As shown in Figure 4b, NFM first undergoes hydrogenation to form NHMM with a Gibbs free energy barrier of 0.37 eV (**TS1'**). The subsequent C─N cleavage (**TS6'**) of NHMM has a Gibbs free energy barrier of 0.96 eV, while the hydrogenative C‐O cleavage has a much higher Gibbs free energy barrier of 1.39 eV (**TS7'**). This indicates that C─N cleavage leading to the subsequent formation of methanol and morpholine is much more favored kinetically than the C‐O cleavage leading to the formation of NMM by 0.43 eV, indicating the higher selectivity. However, it is interesting to note that the direct C─N bond dissociation of NFM has a lower apparent Gibbs free energy barrier (**TS2'**) than the hydrogenative C─N bond dissociation (**TS6'**) by 0.52 eV. These results show that on the supported Pt, the selective formation of morpholine and methanol is kinetically more favored, especially via the direct C─N bond cleavage of NFM.

Comparing these results on Pt(111) and Pt/TiO_2_(101) surfaces reveals that small and well‐dispersed Pt clusters provide excellent selectivity toward morpholine and methanol, while large metallic Pt clusters show poor selectivity. This trend agrees with our experimental results up to quantitative selectivity (Table 1, entries 11–13 vs. 14).

Taken together, these data highlight the interplay between catalyst structure, electronic state, and performance. NFM conversion increases with Pt loading; however, once the loading reaches 2%, Pt aggregates into nanoparticles, reducing the density of accessible active sites. As a result, 2%Pt/P25 shows little improvement in conversion compared to 1%Pt/P25 (Figure 3c). Methanol selectivity, in contrast, is governed primarily by Pt electronic state: catalysts containing predominantly Pt^δ−^ species (0.5% and 1%Pt/P25) sustain nearly 100% methanol selectivity, whereas 2%Pt/P25, dominated by metallic Pt^0^, exhibits lower selectivity (Figure 3c). Balancing conversion and selectivity identifies 1%Pt/P25 as the optimal catalyst.

**FIGURE 5:**
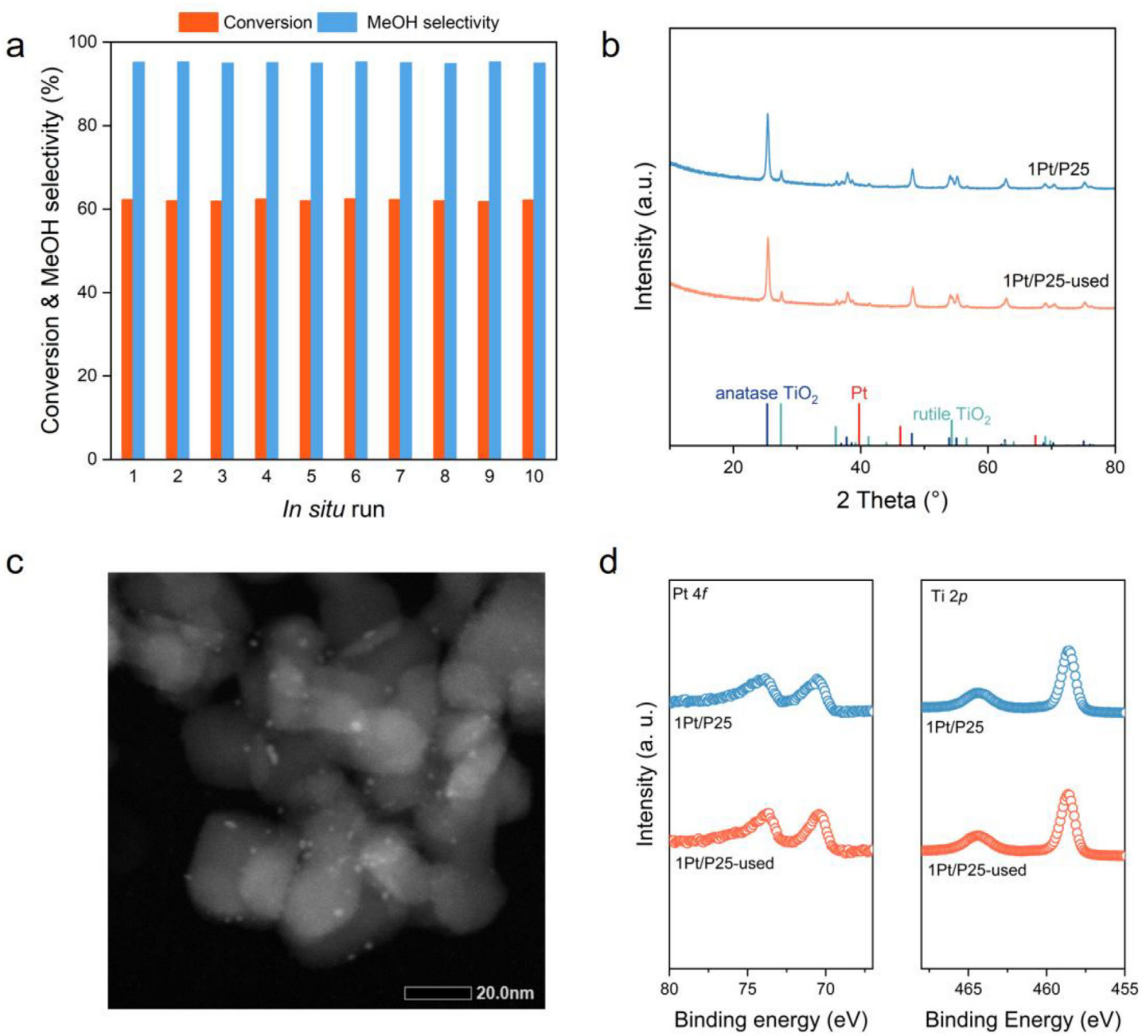
The stability of the 1%Pt/P25 catalyst. (a) In situ recycle performance of the 1%Pt/P25 catalyst over 10 runs. (b) XRD results for the used 1%Pt/P25 catalyst. (c) HAADF‐STEM images for the used 1%Pt/P25 catalyst. (d) In situ XPS of Pt 4*f* and Ti 2*p* spectrum for the used 1%Pt/P25 catalyst.

Notably, this catalyst also displays excellent stability. It maintains consistent activity and selectivity over ten consecutive recycles (Figure 5a). Post‐reaction XRD (Figure 5b) and HAADF‐STEM (Figure 5c and Figures S23 and S24) confirm that the Pt clusters remain intact without detectable sintering, while XPS analysis shows that the Pt^δ–^ character persists (Figure 5d). These results demonstrate that strong metal–support interactions stabilize both the structural and electronic properties responsible for high methanol selectivity.

## 3 Conclusion

In this study, we have identified Pt/P25 catalysts as highly selective systems for the hydrogenation of CO_2_‐derived formamides to methanol, while simultaneously regenerating the CO_2_ sorbent (morpholine) for reuse in capture. The optimal Pt/P25 catalyst achieves up to 62% conversion of 4‐formylmorpholine with ∼95% methanol selectivity at 150°C, outperforming analogous Ru, Pd, Rh, and Ir catalysts, which instead favor undesired C─O bond cleavage to 4‐methylmorpholine. In situ characterizations (HAADF‐STEM, XAS, XPS) reveal that strong metal–support interactions at the Pt–TiO_2_ interface generate electron‐rich Pt^δ–^ species. These sites preferentially promote efficient methanol formation and complete amine regeneration. The catalyst maintains stable performance over ten cycles without sintering or loss of electronic enrichment. This structure–activity relationship highlights the importance of maximizing interfacial Pt–TiO_2_ contacts and tuning Pt electronic properties for selective methanol generation. Furthermore, this work establishes a “mild‐temperature”, integrated CO_2_ capture‐and‐conversion route in a heterogeneous system that directly links amine sorbent cycling with methanol synthesis. Beyond CO_2_ utilization, the electronic control strategy may be extended to amide hydrogenations relevant to chemical and biomass upgrading. Overall, this approach provides a robust platform toward an industrial CO_2_‐to‐methanol conversion with broad implications for advancing a carbon‐neutral fuel economy.

## Conflicts of Interest

The authors declare no conflicts of interest.

## Supporting information




**Supporting File**: anie72560‐sup‐0001‐SuppMat.docx.

## Data Availability

The data that support the findings of this study are available in the Supporting Information of this article.

## References

[1] D. P. Tans , https://gml.noaa.gov/ccgg/trends/.

[2] E. S. Sanz‐Perez , C. R. Murdock , S. A. Didas , and C. W. Jones , “Direct Capture of CO_2_ From Ambient Air,” Chemical Reviews 116 (2016): 11840–11876, 10.1021/acs.chemrev.6b00173. 27560307

[3] A. Goeppert , M. Czaun , G. K. Surya Prakash , and G. A. Olah , “Air as the Renewable Carbon Source of the Future: An Overview of CO_2_ Capture From the Atmosphere,” Energy & Environmental Science 5 (2012): 7833–7853.

[4] K. Z. House , A. C. Baclig , M. Ranjan , E. A. van Nierop , J. Wilcox , and H. J. Herzog , “Economic and Energetic Analysis of Capturing CO_2_ From Ambient Air,” PNAS 108 (2011): 20428–20433, 10.1073/pnas.1012253108. 22143760 PMC3251141

[5] D. Wei , R. Sang , A. Moazezbarabadi , H. Junge , M. Beller , and J. Au , “Homogeneous Carbon Capture and Catalytic Hydrogenation: Toward a Chemical Hydrogen Battery System,” JACS Au 2 (2022): 1020–1031. 35647600 10.1021/jacsau.1c00489 PMC9131476

[6] M. Bui , C. S. Adjiman , A. Bardow , et al., “Carbon Capture and Storage (CCS): The Way Forward,” Energy & Environmental Science 11 (2018): 1062–1176, 10.1039/C7EE02342A.

[7] D. J. Heldebrant , J. Kothandaraman , N. M. Dowell , and L. Brickett , “Next Steps for Solvent‐Based CO_2_ Capture; Integration of Capture, Conversion, and Mineralisation,” Chemical Science 13 (2022): 6445–6456, 10.1039/D2SC00220E. 35756509 PMC9172129

[8] D. J. Heldebrant , J. Kothandaraman , and D. Reiner , Carbon Capture and Storage, ed. M. Bui , N. Mac Dowell (The Royal Society of Chemistry, 2019): 0.

[9] C. Das Neves Gomes , O. Jacquet , C. Villiers , P. Thuery , M. Ephritikhine , and T. Cantat , “A Diagonal Approach to Chemical Recycling of Carbon Dioxide: Organocatalytic Transformation for the Reductive Functionalization of CO_2_ ,” Angewandte Chemie 51 (2012): 187–190, 10.1002/anie.201105516. 21960366

[10] D. U. Nielsen , X.‐M. Hu , K. Daasbjerg , and T. Skrydstrup , “Chemically and Electrochemically Catalysed Conversion of CO_2_ to CO With Follow‐Up Utilization to Value‐Added Chemicals,” Nature Catalysis 1 (2018): 244–254, 10.1038/s41929-018-0051-3.

[11] Q. Liu , L. Wu , R. Jackstell , and M. Beller , “Using Carbon Dioxide as a Building Block in Organic Synthesis,” Nature Communications 6 (2015): 5933, 10.1038/ncomms6933. 25600683

[12] K. Dong , R. Razzaq , Y. Hu , K. Ding , and X.‐F. Wu , “Homogeneous Reduction of Carbon Dioxide With Hydrogen,” in Chemical Transformations of Carbon Dioxide (Springer, 2018), 203–228.

[13] A. Alvarez , A. Bansode , A. Urakawa , et al., “Challenges in the Greener Production of Formates/Formic Acid, Methanol, and DME by Heterogeneously Catalyzed CO_2_ Hydrogenation Processes,” Chemical Reviews 117 (2017): 9804–9838, 10.1021/acs.chemrev.6b00816. 28656757 PMC5532695

[14] L. Zhang , Z. Han , X. Zhao , Z. Wang , and K. Ding , “Highly Efficient Ruthenium‐Catalyzed N‐Formylation of Amines With H_2_ and CO_2_ ,” Angewandte Chemie 54 (2015): 6186–6189, 10.1002/anie.201500939. 25850597

[15] Y. Shen , Q. Zheng , Z. N. Chen , et al., “Highly Efficient and Selective N‐Formylation of Amines With CO_2_ and H_2_ Catalyzed by Porous Organometallic Polymers,” Angewandte Chemie 60 (2021): 4125–4132, 10.1002/anie.202011260. 33200851

[16] D. Cheng , M. Wang , L. Tang , et al., “Catalytic Synthesis of Formamides by Integrating CO_2_ Capture and Morpholine Formylation on Supported Iridium Catalyst,” Angewandte Chemie 61 (2022): e202202654, 10.1002/anie.202202654. 35394704

[17] J. Kothandaraman , A. Goeppert , M. Czaun , G. A. Olah , and G. K. Prakash , “Conversion of CO_2_ From Air Into Methanol Using a Polyamine and a Homogeneous Ruthenium Catalyst,” Journal of the American Chemical Society 138 (2016): 778–781, 10.1021/jacs.5b12354. 26713663

[18] J. Kothandaraman , J. S. Lopez , Y. Jiang , et al., “Integrated Capture and Conversion of CO_2_ to Methanol in a Post‐Combustion Capture Solvent: Heterogeneous Catalysts for Selective C─N Bond Cleavage,” Advanced Energy Materials 12 (2022): 2202369.

[19] H. Zhao , R. Yu , S. Ma , et al., “The Role of Cu_1_–O_3_ Species in Single‐atom Cu/ZrO_2_ Catalyst for CO_2_ Hydrogenation,” Nature Catalysis 5 (2022): 818–831, 10.1038/s41929-022-00840-0.

[20] C. Wu , L. Lin , J. Liu , et al., “Inverse ZrO_2_/Cu as a Highly Efficient Methanol Synthesis Catalyst From CO_2_ Hydrogenation,” Nature Communications 11 (2020): 5767, 10.1038/s41467-020-19634-8. PMC7666171 33188189

[21] X. Jiang , X. Nie , X. Guo , C. Song , and J. G. Chen , “Recent Advances in Carbon Dioxide Hydrogenation to Methanol via Heterogeneous Catalysis,” Chemical Reviews 120 (2020): 7984–8034, 10.1021/acs.chemrev.9b00723. 32049507

[22] J. Kothandaraman , D. J. Heldebrant , J. S. Lopez , and R. A. Dagle , “Mechanistic Insights to Drive Catalytic Hydrogenation of Formamide Intermediates to Methanol via Deaminative Hydrogenation,” Frontiers in Energy Research 11 (2023): 1158499.

[23] E. Balaraman , B. Gnanaprakasam , L. J. Shimon , and D. Milstein , “Direct Hydrogenation of Amides to Alcohols and Amines Under Mild Conditions,” Journal of the American Chemical Society 132 (2010): 16756–16758, 10.1021/ja1080019. 21049928

[24] V. Papa , J. R. Cabrero‐Antonino , E. Alberico , et al., “Efficient and Selective Hydrogenation of Amides to Alcohols and Amines Using a Well‐Defined Manganese–PNN Pincer Complex,” Chemical Science 8 (2017): 3576–3585, 10.1039/C7SC00138J. 30155202 PMC6092716

[25] J. R. Cabrero‐Antonino , R. Adam , V. Papa , and M. Beller , “Homogeneous and Heterogeneous Catalytic Reduction of Amides and Related Compounds Using Molecular Hydrogen,” Nature Communications 11 (2020): 3893, 10.1038/s41467-020-17588-5. PMC7403344 32753681

[26] S. Kar , R. Sen , J. Kothandaraman , et al., “Mechanistic Insights Into Ruthenium‐Pincer‐Catalyzed Amine‐Assisted Homogeneous Hydrogenation of CO_2_ to Methanol,” Journal of the American Chemical Society 141 (2019): 3160–3170, 10.1021/jacs.8b12763. 30753062

[27] A. Moazezbarabadi , A. Kammer , E. Alberico , H. Junge , and M. Beller , “Amino Acid‐Based Ionic Liquids‐Aided CO_2_ Hydrogenation to Methanol,” Chemsuschem 18 (2025): e202401813, 10.1002/cssc.202401813. 39520398 PMC11960586

[28] J. Kothandaraman and D. J. Heldebrant , “Towards Environmentally Benign Capture and Conversion: Heterogeneous Metal Catalyzed CO_2_ Hydrogenation in CO_2_ Capture Solvents,” Green Chemistry 22 (2020): 828–834, 10.1039/C9GC03449H.

[29] R. Kumar , T. Mandal , A. Bhattacherya , M. K. Pandey , J. K. Bera , and J. Choudhury , “Hyper‐Cross‐Linked Polymer‐Based Self‐Supported Reusable Ru‐NHC Catalyst for Amine‐Assisted Hydrogenation of CO_2_ to Methanol,” ACS Catalysis 14 (2024): 13236–13245, 10.1021/acscatal.4c02513.

[30] D. Wen , J. Chen , Q. Zheng , S. Yang , and T. Tu , “Directly Knitted Ruthenium Pincer Complexes With Enhanced Activity as Recyclable Single‐Site Catalysts for Hydrogenation of CO_2_ to Methanol,” CCS Chemistry 2023 5 (2023): 1602–1611, 10.31635/ccschem.022.202202233.

[31] S. J. Tauster , S. C. Fung , and R. L. Garten , “Strong Metal‐Support Interactions. Group 8 Noble Metals Supported on Titanium Dioxide,” Journal of the American Chemical Society 100 (2002): 170–175, 10.1021/ja00469a029.

[32] M. Xu , M. Peng , H. Tang , W. Zhou , B. Qiao , and D. Ma , “Renaissance of Strong Metal–Support Interactions,” Journal of the American Chemical Society 146 (2024): 2290–2307, 10.1021/jacs.3c09102. 38236140

[33] L. Artus Suarez , U. Jayarathne , D. Balcells , et al., “Rational Selection of Co‐Catalysts for the Deaminative Hydrogenation of Amides,” Chemical Science 11 (2020): 2225–2230, 10.1039/C9SC03812D. 32190278 PMC7059200

[34] D. Wei , X. Shi , H. Junge , C. Du , and M. Beller , “Carbon Neutral Hydrogen Storage and Release Cycles Based on Dual‐Functional Roles of Formamides,” Nature Communications 14 (2023): 3726, 10.1038/s41467-023-39309-4. PMC10287642 37349304

[35] M. Tamura , S. Ishikawa , M. Betchaku , Y. Nakagawa , and K. Tomishige , “Selective Hydrogenation of Amides to Alcohols in Water Solvent Over a Heterogeneous CeO_2_‐Supported Ru Catalyst,” Chemical Communications 54 (2018): 7503–7506, 10.1039/C8CC02697A. 29924114

[36] Y. Xie , P. Hu , T. Bendikov , and D. Milstein , “Heterogeneously Catalyzed Selective Hydrogenation of Amides to Alcohols and Amines,” Catalysis Science & Technology 8 (2018): 2784–2788, 10.1039/C8CY00112J.

[37] N. M. Rezayee , C. A. Huff , and M. S. Sanford , “Tandem Amine and Ruthenium‐Catalyzed Hydrogenation of CO_2_ to Methanol,” Journal of the American Chemical Society 137 (2015): 1028–1031, 10.1021/ja511329m. 25594380

[38] M. Everett and D. F. Wass , “Highly Productive CO_2_ Hydrogenation to Methanol—A Tandem Catalytic Approach via Amide Intermediates,” Chemical Communications 53 (2017): 9502–9504, 10.1039/C7CC04613H. 28661540

[39] S. Kar , J. Kothandaraman , A. Goeppert , and G. K. S. Prakash , “Advances in Catalytic Homogeneous Hydrogenation of Carbon Dioxide to Methanol,” Journal of CO2 Utilization 23 (2018): 212–218, 10.1016/j.jcou.2017.10.023.

[40] X. Wu , W. T. Lee , R. C. Turnell‐Ritson , P. C. L. Delannoi , K. H. Lin , and P. J. Dyson , “Controlling the Selectivity of the Hydrogenolysis of Polyamides Catalysed by Ceria‐Supported Metal Nanoparticles,” Nature Communications 14 (2023): 6524, 10.1038/s41467-023-42246-x. PMC10579319 37845260

[41] X. Sun , X. Shen , H. Wang , et al., “Atom‐Level Interaction Design Between Amines and Support for Achieving Efficient and Stable CO_2_ Capture,” Nature Communications 15 (2024): 5068, 10.1038/s41467-024-48994-8. PMC11176289 38871697

[42] K. Motokura , M. Tada , and Y. Iwasawa , “Cooperative Catalysis of Primary and Tertiary Amines Immobilized on Oxide Surfaces for One‐Pot C—C Bond Forming Reactions,” Angewandte Chemie 47 (2008): 9230–9235, 10.1002/anie.200802515. 18767097

[43] J. van Bokhoven , “Recent Developments in X‐Ray Absorption Spectroscopy,” Physical Chemistry Chemical Physics 12 (2010): 5502. 20473425 10.1039/c0cp90010a

[44] A. Iglesias‐Juez , G. L. Chiarello , G. S. Patience , and M. O. Guerrero‐Pérez , “Experimental Methods in Chemical Engineering: X‐Ray Absorption Spectroscopy—XAS, XANES, EXAFS,” Canadian Journal of Chemical Engineering 100 (2021): 3–22, 10.1002/cjce.24291.

[45] Z. Gao , L. Cai , C. Miao , et al., “Electronic Metal‐Support Interaction Strengthened Pt/CoAl‐LDHs Catalyst for Selective Cinnamaldehyde Hydrogenation,” Chemcatchem 14 (2022): e202200634.

[46] J. Chen , H. Weng , Z. Li , et al., “Spotlight on Pt/γ‐Al_2_O_3_ With High Catalytic Performance Induced by Barium: Synergistic effect of Electron‐Rich Ptδ‐Single‐Atoms and Available Oxygen Species,” Chemical Engineering Journal 474 (2023): 145574.

