# Supplementary material for: Selective Hydrogenation of Formamide to Methanol Over Supported Platinum Catalysts
Source: Angew Chem Int Ed Engl. 2026 May 8;65(25):e22476. doi: 10.1002/anie.202522476 (PMC13266920; doi:10.1002/anie.202522476)
Supplement: Supplementary file 1 — Supporting File: anie72560‐sup‐0001‐SuppMat.docx. [file ANIE-65-e22476-s001.docx]

Supplementary Materials for

Selective Hydrogenation of Formamide to Methanol over Supported Platinum Catalysts

Xuetao Qin^[a]^†, XinXin Tian^[a,b]^†, Shou Qiu^[c]^, Han Yan^[d]^, Haijun Jiao^[a]^*, Henrik Junge^[a]*^, Matthias Beller^[a]*^

[a]	Dr. X. Qin, Dr. X. Tian, Prof. Haijun Jiao, Dr. H. Junge, Prof. Dr. M. Beller
Leibniz-Institut für Katalyse e.V., Albert-Einstein-Str. 29a, Rostock, D-18059, Germany
E-mail: [haijun.jiao@catalysis.de](mailto:haijun.jiao@catalysis.de); [henrik.junge@catalysis.de](mailto:henrik.junge@catalysis.de); matthias.beller@catalysis.de

[b]	Dr. X. Tian

Institute of Molecular Science, Shanxi University, Taiyuan 030006, China

[c]	S. Qiu
Beijing National Laboratory for Molecular Engineering, New Cornerstone Science Laboratory, College of Chemistry and Molecular Engineering, Peking University, Beijing 100871, P. R. China

[d]	Dr. H. Yan
Hefei National Research Center for Physical Sciences at the Microscale, Key Laboratory of Strongly-Coupled Quantum Matter Physics of Chinese Academy of Sciences, Key Laboratory of Surface and Interface Chemistry and Energy Catalysis of Anhui Higher Education Institutes, Department of Chemical Physics, University of Science and Technology of China, Hefei, People’s Republic of China

†These authors contributed equally to this work

**The PDF file includes:**

Materials and Methods

Figs. S1 to S37

Tables S1 to S5

References

**Materials and Methods**

Catalyst preparation

1. **Metal supported on carbon support**

Catalysts were synthesized by impregnation method. For 1%Pt/C catalyst, H_2_PtCl_6_·*x*H_2_O (Sigma-Aldrich) was mixed with the commercial carbon black (Vulcan XC-72, Sigma-Aldrich) support (1 g) using a wetness impregnation method. Specifically, 1 g of carbon black support was dispersed in 20 mL of deionized water at first, and appropriate amounts of a H_2_PtCl_6_·*x*H_2_O aqueous solution were added dropwise to obtain the desired catalyst. The suspension liquid was stirred for 1 h. The solvent (water) was removed by rotary evaporation. The solid was dried at 60°C in vacuum, then calcined at 400°C under Ar with a 5°C/min ramping rate. Next, the as-obtained mixed oxides were reduced in a H_2_/Ar (5:95, v/v) stream for 2 h at 200°C with a flow rate of 50 mL/min and a heating rate of 2 °C/min. After the reduction process, air was leaked onto the catalyst surface, passivating the catalyst overnight. For other metals (RuCl_3_·*x*H_2_O, Sigma-Aldrich; RhCl_3_·*x*H_2_O, Sigma-Aldrich; K_2_PdCl_4_, Sigma-Aldrich; H_2_IrCl_6_·*x*H_2_O, Sigma-Aldrich) supported on carbon support were synthesized by the same method.

2. **1%Pt supported on various supports**

Catalysts were synthesized by the same impregnation method. For 1%Pt/C catalyst, H_2_PtCl_6_·*x*H_2_O (Sigma-Aldrich) was mixed with the commercial carbon black (Vulcan XC-72, Sigma-Aldrich) support (1 g) using a wetness impregnation method. Specifically, 1 g of carbon black support was dispersed in 20 mL of deionized water at first, and appropriate amounts of a H_2_PtCl_6_·*x*H_2_O aqueous solution were added dropwise to obtain the desired catalyst. The suspension liquid was stirred for 1 h. The solvent (water) was removed by rotary evaporation. The solid was dried at 60°C in vacuum, then calcined at 400°C in Ar with a 5°C/min ramping rate. Next, the as-obtained mixed oxides were reduced in a H_2_/Ar (5:95, v/v) stream for 2 h at 200°C with a flow rate of 50 mL/min and a heating rate of 2 °C/min. After reduction process, the air was leaked onto the catalyst surface, passivating the catalyst overnight. For 1%Pt supported on other support (CeO_2_, Sigma-Aldrich; Al_2_O_3_, Sigma-Aldrich; H-ZSM-5, Sigma-Aldrich; MgO, Sigma-Aldrich; P25 Sigma-Aldrich) were synthesized by the same method.

3. ***x*%Pt supported on P25**

Catalysts were synthesized by the same impregnation method. For 1%Pt/P25 catalyst, H_2_PtCl_6_·*x*H_2_O (Sigma-Aldrich) was mixed with the commercial P25 (Sigma-Aldrich) support (1 g) using a wetness impregnation method. Specifically, 1 g of carbon black support was dispersed in 20 mL of deionized water at first, and appropriate amounts of a H_2_PtCl_6_·*x*H_2_O aqueous solution were added dropwise to obtain the desired catalyst. The suspension liquid was stirred for 1 h. The solvent (water) was removed by rotary evaporation. The solid was dried at 60°C in vacuum, then calcined at 400°C in Ar with a 5°C/min ramping rate. Next, the as-obtained mixed oxides were reduced in a H_2_/Ar (5:95, v/v) stream for 2 h at 200°C with a flow rate of 50 mL/min and a heating rate of 2 °C/min. After reduction process, the air was leaked onto the catalyst surface, passivating the catalyst overnight. For *x*%Pt supported on P25 were synthesized by the same method.

Catalytic performance evaluation

The catalytic hydrogenation reactions were conducted in a 100 mL autoclave. Typically, 100 mg catalyst was set into the autoclave with 30 mL solvent. The sealed autoclave was purged using N_2_ three times and using H_2_ three times. Then, the autoclave was purged 20 bar hydrogen. The catalyst was pre-treated in 20 bar hydrogen at 200 °C for one hour to activate the passivated catalyst. After the catalysts were reduced in the autoclave, released the hydrogen and added 20 mmol *N*-formyl morpholine into the autoclave. The sealed autoclave was purged using N_2_ three times and using H_2_ three times. Then, the autoclave was purged 50 bar hydrogen. The catalytic reactions were carried out at various reaction conditions.

The liquid products were characterized by Gas chromatography–mass spectrometry (GC–MS), ^1^H NMR and ^13^C NMR spectroscopy. The NMR spectra were recorded on Bruker Avance 300 (300 MHz) or 400 (400 MHz) NMR spectrometers. MestReNova (version 14.0.1-23559) was used for interpreting and processing the NMR spectra. The ^1^H NMR were calibrated against the peak of DMSO at 2.71 ppm. The ^13^C NMR were calibrated against the peak of the DMSO at 39.39 ppm.

GC-MS analysis: Gas chromatography–mass spectrometry (GC–MS) measurements were carried out on an Agilent 8890 GC system equipped with an HP-5MS UI capillary column and an Agilent 5977C GC/MSD detector. After reaction, the catalyst was removed by filtration. A 50 µL aliquot of the filtrate was transferred into a GC vial and diluted with acetone prior to analysis. The diluted sample was then injected for GC–MS measurement (Fig. S1).

Sample preparation for quantitative NMR: After the reaction, the liquid reaction mixture was filtered to remove any solid catalyst or particulates. A 1 mL aliquot of the filtered solution was then taken, to which 100 µL of deuterium oxide (D_2_O) and 100 µL of dimethyl sulfoxide (DMSO) were added. The D_2_O provides a deuterium lock signal for the NMR instrument, and DMSO was added as an internal standard of known quantity. The mixture was homogenized and transferred to an NMR tube for analysis. The ^1^H NMR spectra of the standard sample and reaction products is provided (Figs. S2-6).

The formula used for calculation of the mole of product is:

𝑛MeOH=𝑛DMSO×(𝐼MeOH/3)(𝐼DMSO/6),

where 𝑛MeOH is the number of moles of methanol in the NMR sample, 𝑛DMSO is the number of moles of DMSO added (internal standard), 𝐼MeOH is the integrated area of the methanol ^1^H signal (accounting for 3 protons per CH_3_ group), and 𝐼DMSO is the integrated area of the DMSO ^1^H signal (accounting for 6 protons total for DMSO's two CH_3_ groups). Using this ratio of normalized integrals ensures accurate quantification of methanol relative to the internal standard. *N*-formyl morpholine (NFM), *N*-methyl morpholine (NMM) and morpholine (M) are quantified to obtain 𝑛NFM, 𝑛NMM and 𝑛M according to this same method.

The formula used for reaction calculation is:

$$Conversion=\frac{nNFMintial-nNFMfinal}{nNFMintial}\times100\%$$

$$Selectivity(M)=\frac{nM}{nM+nNMM}\times100\%$$

$$Selectivity(NMM)=\frac{nNMM}{nM+nNMM}\times100\%$$

$$Selectivity(MeOH)=\frac{nMeOH}{nM+nNMM}\times100\%$$

$$Carbon balance=\frac{nNFMfinal+nNMM+nMeOH}{nNFMintial}\times100\%$$

Where 𝑛NFMintial is the number of moles of NFM added to the reaction, 𝑛NFMfinal is the number of moles of NFM after the reaction, 𝑛NMM is the number of moles of NMM after the reaction, 𝑛M is the number of moles of M after the reaction.

Stability evaluation

The stability of the 1%Pt/P25 catalyst was evaluated using the same reactor as used for catalytic performance tests. After each cycle of hydrogenation reaction and product analysis, the spent catalyst and reaction solution were separated by rotary evaporation. And the spent catalyst was then washed with 1,4-dioxane for three times. During the rotary evaporation and washing procedure, the spent catalyst should be submerged below the liquid level as far as possible, so as to avoid the contact between the catalyst and air. Following this procedure, 20 mmol of *N*-formyl morpholine, solvent and 50 bar of H_2_ were introduced for each of the ten recycling experiments.

Characterization of catalysts

1. **X-ray Diffraction (XRD)**

Powder XRD experiments were carried out on a X-Pert^3^ Powder diffractometer with Cu *K*α radiation (Condition: 40 kV, and 100 mA; Scan rate: 5°min^−1^) and a 2*θ* angle ranging from 5° to 80°.

2. **X-ray photoelectron spectroscopy (XPS)**

XPS measurements were conducted on an Axis Ultra Imaging Photoelectron Spectrometer equipped with an Al *K*α quartz monochrometer source. Quasi-in situ XPS results of the reduced catalysts were measured by transferring the samples to the measurement chamber without exposure to air. The binding energy was corrected by setting the C 1*s* signal of 284.8 eV as the reference. All XPS spectra were processed using CasaXPS software.

3. **X-ray absorption spectroscopy (XAS)**

The XAS spectra of Pt *L_3_*-edge (11564 eV) were collected at 1W1B beamline of Beijing Synchrotron Radiation Facility (BSRF), Institute of High Energy Physics (IHEP), Chinese Academy of Sciences (CAS). The beam was tuned by the Si (111) double-crystal monochromators. The energies were calibrated according to the absorption edge of Pt foil. The typical energy of the storage ring was 2.5 GeV with a maximum current of 250 mA. For quasi in situ XAS spectra of the reduced catalysts, the powdered sample was pressed into the sheet in the glovebox without exposure to air.

4. **Transmission Electron Microscopy (TEM)**

High-angle annular dark-field imaging (HAADF) and high-resolution transmission electron microscopy (HRTEM) experiments were performed on a JEOL JFM-F200 electron microscope equipped with a probe-forming aberration corrector operated at an accelerating voltage of 200 kV. The samples were ultrasonically dispersed in ethanol and then dropped and dried on copper grids with holey carbon.

Figure S1. GC-MS results regarding to blank acetone (a), Table 1 Entry 11 (b) and Table 1 Entry 14 (c).

Figure S2. ^1^H NMR (a) and ^13^C NMR (b) of *N*-formylmorpholine with DMSO.

Figure S3. ^1^H NMR (a) and ^13^C NMR (b) of morpholine with DMSO.

Figure S4. ^1^H NMR (a) and ^13^C NMR (b) of *N*-methylmorpholin with DMSO.

Figure S5. ^1^H NMR (a) and ^13^C NMR (b) of methanol with DMSO.

Figure S6. ^1^H NMR (a) and ^13^C NMR (b) of Table 1 Entry 11 with DMSO.

Figure S7. HADDF-STEM images of 0.1%Pt/P25 with various scales.

Figure S8. HRTEM images of 0.1%Pt/P25 with various scales.

Figure S9. HADDF-STEM images of 0.5%Pt/P25 with various scales.

Figure S10. HRTEM images of 0.5%Pt/P25 with various scales.

Figure S11. HADDF-STEM images of 1%Pt/P25 with various scales.

Figure S12. HRTEM images of 1%Pt/P25 with various scales.

Figure S13. HADDF-STEM images of 2%Pt/P25 with various scales.

Figure S14. HRTEM images of 2%Pt/P25 with various scales.

Figure S15. Wavelet transform of Pt *L*_3_-edge XAS for PtO_2_.

Figure S16. Wavelet transform of Pt *L*_3_-edge XAS for Pt foil.

Figure S17. Wavelet transform of Pt *L*_3_-edge XAS for 0.1%Pt/P25.

Figure S18. Wavelet transform of Pt *L*_3_-edge XAS for 0.5%Pt/P25.

Figure S19. Wavelet transform of Pt *L*_3_-edge XAS for 1%Pt/P25.

.

Figure S20. Wavelet transform of Pt *L*_3_-edge XAS for 2%Pt/P25.

Figure S21. EXAFS fitting results of various *x*%Pt/P25 catalysts.

Figure S22. Quasi in situ XPS spectra of Ti 2*p*, O 1*s* and C 1*s* for catalysts

Figure S23. HADDF-STEM images of 1%Pt/P25-used.

Figure S24. HRTEM images of 1%Pt/P25-used with various scales.

**Computational methods and models**

**1. Methods**

Spin-polarized periodic density functional theory (DFT) calculations were carried out with the Vienna *ab initio* simulation package (VASP).^[5]^ The electron exchange and correlation energies were treated using the generalized gradient approximation in the Perdew-Burke-Ernzerhof (GGA-PBE) functional.^[6]^ The cut-off energy was set up to 400 eV. Geometry optimization was performed using PBE under the consideration of van der Waals dispersion correction using the latest parameters (GGA-PBE-D3)^[7]^ and converged until forces acting on atoms lower than 0.02 eV/Å, and energy difference lower than 10^−5^ eV. The Hubbard correction (DFT+*U*_eff_)^[8]^ was applied to interface models. The effective Hubbard parameter *U*_eff_ was specified for the 3*d* orbitals of Ti (4.5). The Climbing Image Nudged Elastic Band (CI-NEB) method was applied for identifying transition states.^[9]^ Gibbs free energy correction at the given temperature was made by using the VASPKIT program.^[10]^

**2. Models**

To clarify the intrinsic activity of catalysts, DFT calculations were performed on two models (Fig. S25). A *p*(2×2) supercell with four atomic layers for Pt(111) surface was used to simulate the large crystalline metallic Pt particles observed on the catalyst with high Pt loading. A *p*(1×3) supercell with three stoichiometric layers for TiO_2_(101) surface (anatase phase) terminated by co-exposed Ti and O atoms was used as the support based on the XRD characterization results. To simulate the strong interaction between small-sized Pt nanoparticles and the TiO_2_ support for the catalyst with low Pt loading as well as the effect of support on the Pt valence state, the six Ti atoms in the outermost atomic layer were replaced with Pt atoms, and an additional layer of Pt atoms was subsequently deposited on the TiO_2_(101) surface. This model yields an average Pt-Pt coordination number of 4 and an average Pt-O coordination number of 0.92 (considering only Pt atoms coordinated to O, the average coordination number is 3.67, close to 4). The charge range of Pt atoms is between -0.15 *e* and 0.95 *e*, with the outermost surface Pt atoms having a charge range of -0.15 *e* to 0.04 *e* and an average charge of -0.04 *e*. These results are consistent with experimental EXAFS characterizations of Pt properties. Therefore, we considered this model, Pt/TiO_2_(101), to be reasonable.

Figure S25. Top and side views of Pt(111) and Pt/TiO_2_(101) models. Light gray, red, and cyan spheres represent Ti, O, and Pt atoms, respectively.

**3. Possible reaction pathways of *N*-formyl morpholine (NFM) on Pt(111) surface**

To gain an overall understanding of the reaction mechanism, we first explored various possible reaction pathways of NFM on the Pt(111) surface. Initially, we compared multiple isomers of NFM adsorbed on the surface and found that the most stable adsorption configuration involves the O atom of the formyl group interacting with a Pt surface atom, forming a Pt-top adsorption configuration. The adsorption energy for this configuration is -0.41 eV. Meanwhile, the configuration where C, N, and O atoms interact with the surface is less stable with adsorption energy of -0.26 eV. The corresponding adsorption configurations and related bond lengths are shown in Fig. S26. The same energetic order is also found for the *N*-hydroxy(morpholino)methyl (NHMM), the single site adsorption configuration is more stable than the triple site adsorption configuration (-0.71 vs. 0.63 eV). The Bader charges of C, N, O, and H atoms in the gas-phase molecule of NFM, and NFM/NHMM adsorption isomers on the Pt(111) surface are listed in Table S5.

Figure S26. The most stable and second-most stable adsorption configurations of NFM and NHMM on the Pt(111) surface. White, gray, blue, red, and cyan spheres represent H, C, N, O, and Pt atoms, respectively.

Starting from these two adsorption configurations, several possible reaction pathways for NFM were identified, i.e., -CH=O hydrogenation to CH-OH (**P1**, Fig. S27), direct cleavage of C-N bond (**P2**, i.e., removal of the CHO group), direct cleavage of the acyl C=O bond (**P3**, i.e., removal of O), and -CH=O hydrogenation to -CH_2_-O (**P4**). The potential energy surfaces for these pathways are shown in Fig. S28. The structures of the corresponding intermediates and transition-states are depicted in Fig. S29.

Figure S27. Possible reaction pathways of NFM on the Pt(111) surface (blue indicates favorable, red indicates unfavorable).

Figure S28. Potential energy diagram of reaction pathways **P1-P4** on the Pt(111) surface.

Figure S29. The structures of intermediate and transition-states of **P1-P4** pathways on the Pt(111) surface.

Among these possibilities, -CH=O hydrogenation to CH-OH (**P1**) occurs very easily with an elementary-step energy barrier of only 0.27 eV and a potential energy surface barrier position of -0.58 eV (**TS1**). The direct cleavage of the C–N bond (**P2**) has a moderate energy barrier of 0.39 eV, with a potential energy surface position of 0.13 eV (**TS2**). In contrast, the direct C=O deoxygenation process (**P3**) is very difficult, featuring a barrier as high as 2.41 eV and a potential energy surface position of 2.15 eV (**TS3**). Although -CH=O hydrogenation to CH_2_-O (**P4**) exhibits an apparent energy barrier (0.14 vs. 0.13 eV) close to that of C–N bond cleavage (**P2**), its elementary-step energy barrier is 0.70 eV (**TS4**), which is 0.31 eV higher than that of **TS2**. It is evident that **P2** is far more favorable than **P3** and **P4**. Comparing these four possible initial reaction pathways, **P1** is most favored not kinetically but also thermodynamically, while **P2** may also occur due to its low energy barrier, **P3** and **P4** are relatively challenging. Therefore, we further focused on only **P1** and **P2**,while discarded **P3** and **P4**.

Based on **P1**, NHMM can undergo further reactions via three distinct pathways: C-OH bond cleavage to release -OH (**P5**), N-CHOH bond cleavage to release -CHOH group (**P6**), and hydrogenative dehydration to release methylene 4-morpholino-iummethanide and H_2_O (**P7**). The potential energy surfaces for these steps are shown in Fig. S30, and the intermediate and transition-state structures depicted in Fig. S31.

Figure S30. Potential energy diagram of reaction pathways **P5-P7** on the Pt(111) surface.

Figure S31. The structures of intermediate and transition-states of **P5-P7** pathways on the Pt(111) surface.

Computational results indicate that the energy barrier for the C-OH bond cleavage is 1.46 eV, with a potential energy surface position of 0.83 eV (**TS5**). The energy barrier for the N-CHOH bond cleavage is 0.63 eV, with a potential energy surface position of 0.00 eV (**TS6**), while the hydrogenative dehydration process has an energy barrier of 1.03 eV, also at a potential energy surface position of 0.00 eV (**TS7**). It is evident that the **P5** is relatively more difficult due to the high barrier, whereas the **P6** and **P7**, sharing the same apparent energy barriers, are competitive.

The product of the N-CHOH bond cleavage (**P6**) is morpholin-4-ide, which can further undergo hydrogenation to form morpholine via pathway **P8** (Fig. S32). This process has a very low energy barrier of 0.35 eV, with a potential energy surface position at -0.21 eV (**TS8**). The hydrogenative dehydration process (**P7**) yields the *N*-methylenemorpholin-4-ium structure, which can undergo sequential hydrogenations via pathways **P9** and **P10** to form *N*-methylmorpholine (NMM). The energy barrier for these two hydrogenation steps is 0.76 eV and 1.05 eV, respectively, with potential energy surface positions at -0.83 eV and -0.66 eV, respectively. It is evident that the hydrogenation processes occur at relatively lower energy positions on the overall potential energy surfaces.

Figure S32. The structures of intermediate and transition-states of **P8-P10** pathways on the Pt(111) surface.

The overall potential energy surface for NFM reactions on Pt(111) is shown in Fig. S33. After adsorption, NFM tends to undergo -CH=O hydrogenation to form NHMM, with the lowest activation barrier among all pathways. NHMM can then cleave the N-CHOH bond (**P6**) to release -CHOH and generate morpholin-4-ide, followed by hydrogenation to yield the target product morpholine. Alternatively, NHMM can undergo hydrogenative dehydration (**P7**) to produce the side product NMM. The transition states for these pathways are both at the highest point on the potential energy surface (0.00 eV), while subsequent hydrogenation occurs at lower energy positions. Therefore, we can deduce that the selectivity between these pathways is determined by the apparent energy barriers of **P6** and **P7** based on Curtin–Hammett principle. Since both **P6** and **P7** have the same apparent Gibbs free energy barriers, they are not selective for pure metallic Pt catalyst.

Figure S33. Potential energy diagram of NFM possible reaction pathways on the Pt(111) surface.

**4. Possible reaction pathways of *N*-formyl morpholine (NFM) on Pt/TiO_2_(101) surface**

Based on our investigation of the reaction mechanism of NFM on the Pt(111) surface, we propose that NFM first undergoes CH=O hydrogenation to form NHMM (**P1**) on the surface. Subsequently, NHMM undergoes N-CHOH bond cleavage (**P6**) to remove the -CHOH group, and subsequently then proceeds through further hydrogenation to yield the target product Morpholine. If NHMM follows a hydrogenative dehydration process (**P7**), it can produce the byproduct NMM after subsequent hydrogenation steps. Product selectivity is primarily determined by apparent Gibbs free energy barriers of **P6** and **P7**. Additionally, since the energy barrier for direct N-C bond cleavage of NFM (**P2**) to remove the CHO group is also relatively low, we included this possibility for comparison.

Based on these considerations, we have focused our study on these four key steps on the Pt/TiO₂(101) surface to evaluate the product selectivity. Similarly, we begin by examining the adsorption of NFM on the Pt/TiO_2_(101) surface. As this surface exhibits Pt atoms with different oxidation states, Bader charge analysis results are shown in Fig. S34.

Figure S34. Bader charge distribution of surface Pt atoms on the Pt/TiO_2_(101) surface.

Calculations reveal that the most stable adsorption configuration for NFM has the O atom of the formyl group interacting with a surface Pt atom. More importantly, the Pt atom is partially positive charged (+0.03*e*, labeled as **Pt1**), and the adsorption energy is -0.22 eV. The second most stable adsorption structure involves simultaneous interactions between C, N, and O atoms with the surface Pt atoms. Here, the C atom interacts with a Pt atom (**Pt2**) carrying a partial negative charge (-0.13*e*), while the N atom interacts with another Pt atom (**Pt3**) also carrying a partial negative charge (-0.03*e*). The O atom remains interacting with **Pt1**. The adsorption energy is 0.16 eV. The adsorption configurations and related bond lengths are illustrated in Fig. S35. The same energetic order is also found for the *N*-hydroxy(morpholino)methyl (NHMM), the single site adsorption configuration is more stable than the triple site adsorption configuration (-0.05 vs. 0.24 eV). The Bader charges of C, N, O, and H atoms in NFM/NHMM adsorption isomers on the Pt/TiO_2_(101) surface are listed in Table S5.

Figure S35. The most stable and second-most stable adsorption configurations of NFM and NHMM on the Pt/TiO_2_(101) model surface.

Starting from these two stable adsorption configurations, the structures of the intermediates and transition states are computed (Fig. S36) and the reaction potential energy surfaces are shown in Fig. S37. Like that on the Pt(111) surface, NFM first undergoes hydrogenation on the surface to form NHMM (**P1'**), with an energy barrier of 0.65 eV and a potential energy surface position of 0.15 eV (**TS1'**). For NHMM undergoing N-CHOH bond cleavage (**P6'**) to remove the -CHOH group, the energy barrier is 0.50 eV, and the potential energy surface position is 0.74 eV (**TS6'**). Meanwhile, the energy barrier for NHMM hydrogenative dehydration is 1.62 eV, with a potential energy surface position of 1.17 eV (**TS7'**). Interestingly, the energy barrier for NFM undergoing direct C-N bond cleavage (**P2'**) to remove the CHO group is only 0.07 eV, and the potential energy surface position is 0.22 eV (**TS2'**).

Figure S36. The structures of intermediate and transition-states of NFM possible reaction pathways on the Pt/TiO_2_(101) surface.

The most favorable C-N dissociation pathway is **P2'**, where NFM undergoes direct dissociation with an apparent energy barrier of only 0.22 eV, ultimately yielding the target product morpholine. On the other hand, the pathway leading to the byproduct NMM involves **P1'**→**P7'** and subsequent hydrogenation processes, with an apparent energy barrier of 1.17 eV. It is evident that on the Pt/TiO_2_(101) surface, C-N bond dissociation is much easier, with a significantly lower energy barrier compared to dehydration (including C-O bond cleavage) for byproduct formation. Therefore, product selectivity is predominantly toward morpholine. This is also consistent with the experimental results (Table 1, entries 11-13), where product exhibits nearly quantitative selectivity.

Figure S37. Potential energy diagram of NFM possible reaction pathways on the Pt/TiO_2_(101) surface.

Table S1. Comparison of the performance of our and state-of-the-art catalysts.

| Catalyst | Reactants | Conditions | Additives | Conversion  (%) | MeOH selectivity  (%) | MeOH  yield (%) | Ref. |
| --- | --- | --- | --- | --- | --- | --- | --- |
| RuPNN |   *N*-formyl morpholine | 110°C, 32h, THF, 10 atm H_2_ | - | - | - | 97 | 1 |
| MnPNN |   *N*-cyclohexyl formamide | 100°C, 16h, cyclohexane, 30 bar H_2_ | KO*^t^*Bu | >99 | >99 | >99 | 2 |
| RuPNP |   *N*-formyl  piperidine | 145°C, 8h, triglyme, 20 bar H_2_ | K_3_PO_4_ | 74 | - | 79 | 3 |
| Cu/Zn/Al_2_O_3_ |   *N*-formyl  piperidine | 170°C, 12h, THF, 60 bar H_2_ | KO*^t^*Bu | 49 | 50 | 24 | 4 |
| 5Ag/Al_2_O_3_ |   *N*-formyl piperidine | 170°C, 12h, THF, 60 bar H_2_ | KO*^t^*Bu | 9 | 86 | 8 |  |
| 5Pd/Al_2_O_3_ | *N*-formylpiperidine | 170°C, 12h, THF, 60 bar H_2_ | KO*^t^*Bu | 7 | 75 | 5 |  |
| 5Pd/Zn/Al_2_O_3_ |   *N*-formyl piperidine | 170°C, 12h, THF, 60 bar H_2_ | KO*^t^*Bu | 5 | 77 | 4 |  |
| 1Pt/P25 |   N-formyl morpholine | 150°C, 12h, 1,4-dioxane, 50 bar H_2_ | - | 62 | 95 | 59 | this work |

Table S2. Solvent screening for hydrogenation of *N*-formylmorpholine.

| Solvent | Conversion/% | Selectivity/% | | |
| --- | --- | --- | --- | --- |
|  |  | 1 | 2 | 3 |
| 1,4-dioxane | 62.3 | 100.0 | 95.2 | n.d. |
| Toluene | 23.8 | 100.0 | 95.5 | n.d. |
| THF | 28.2 | 100.0 | 94.8 | n.d. |
| *n*-Hexane | 15.6 | 89.0 | 84.6 | 11.0 |

Reaction conditions: 100 mg 1%Pt/TiO_2_, 20 mmol *N*-formylmorpholine, 50 bar H_2_, 30 mL solvent, 150°C, 12h.

Table S3. Temperature screening for hydrogenation of *N*-formylmorpholine.

| Temp./°C | Conversion/% | Selectivity/% | | |
| --- | --- | --- | --- | --- |
|  |  | 1 | 2 | 3 |
| 120 | 9.3 | 100.0 | 93.6 | n.d. |
| 130 | 16.8 | 100.0 | 95.5 | n.d. |
| 150 | 62.3 | 100.0 | 95.2 | n.d. |

Reaction conditions: 100 mg 1%Pt/TiO_2_, 20 mmol *N*-formyl morpholine, 50 bar H_2_, 30 mL 1,4-dioxane, 12h.

Table S4. Curve-fit Parameters for Pt *L*_3_-edge EXAFS.

| Sample^a^ | Shell | d (Å)^b^ | C. N.^c^ | σ^2^ (Å^2^)^d^ | *E*_0_ shift(eV) | R-factor (*10^-3^) |
| --- | --- | --- | --- | --- | --- | --- |
| Pt foil | Pt-Pt | 2.76 | 12 | 0.005 | 6.9 | 1.1 |
| PtO_2_ | Pt-O | 2.01 | 6 | 0.003 | 9.3 | 13.6 |
| 0.1Pt/P25 | Pt-O | 2.04 | 4.1 | 0.008 | 11.1 | 13.3 |
|  | Pt-Pt | 2.75 | 4.0 | 0.002 |  |  |
| 0.5Pt/P25 | Pt-O | 1.98 | 3.0 | 0.003 | 8.6 | 6.1 |
|  | Pt-Pt | 2.74 | 5.1 | 0.002 |  |  |
| 1Pt/P25 | Pt-O | 2.02 | 1.7 | 0.004 | 6.3 | 17.3 |
|  | Pt-Pt | 2.74 | 6.5 | 0.008 |  |  |
| 2Pt/P25 | Pt-O | 2.01 | 0.9 | 0.004 | 5.6 | 7.0 |
|  | Pt-Pt | 2.75 | 7.4 | 0.007 |  |  |

a: The data ranges used in these fittings are 3.0 ≤ *k* ≤ 12.0 Å^–1^ and 1.0 ≤ *R* ≤ 3.0 Å. *S*_0_^2^ was fixed at 0.816, obtained from the Pt foil measured at the same time.

b: The coordination distance.

c: Average coordination number.

d: Debye-Waller factor.

Table S5. Bader charges of C, N, O, and H atoms in the gas-phase molecule of NFM, and NFM/NHMM adsorption isomers on Pt(111), and Pt/TiO_2_(101) surfaces, as well as the adsorption energies (*E*_ads_, eV) of these isomers.

|  |  | Unit:*e* | C | N | O | H | *E*_ads_ (eV) |
| --- | --- | --- | --- | --- | --- | --- | --- |
|  | Gas |  | 1.31 | -1.13 | -1.09 | 0.09 |  |
|   NFM | Pt(111) | NFM-isomer1 | 1.25 | -1.15 | -1.05 | 0.18 | -0.41 |
|  |  | NFM-isomer2 | 0.75 | -0.88 | -0.95 | 0.16 | -0.26 |
|  |  | NHMM-isomer1 | 1.16 | -1.17 | -1.11 | 0.21 | -0.71 |
|  |  | NHMM-isomer2 | 0.64 | -0.87 | -1.15 | 0.16 | -0.63 |
|   NHMM | Pt/TiO_2_(101) | NFM-isomer1 | 1.29 | -1.16 | -1.05 | 0.16 | -0.22 |
|  |  | NFM-isomer2 | 0.81 | -0.88 | -0.98 | 0.11 | 0.16 |
|  |  | NHMM-isomer1 | 1.27 | -1.15 | -1.11 | 0.18 | -0.05 |
|  |  | NHMM-isomer2 | 0.57 | -0.89 | -1.06 | 0.11 | 0.24 |

**References**

[1]	E. Balaraman, B. Gnanaprakasam, L. J. Shimon, D. Milstein, J Am Chem Soc 2010, 132, 16756–16758.

[2]	V. Papa, J. R. Cabrero-Antonino, E. Alberico, A. Spanneberg, K. Junge, H. Junge, M. Beller, Chem Sci 2017, 8, 3576–3585.

[3]	S. Kar, R. Sen, J. Kothandaraman, A. Goeppert, R. Chowdhury, S. B. Munoz, R. Haiges, G. K. S. Prakash, J Am Chem Soc 2019, 141, 3160–3170.

[4]	J. Kothandaraman, D. J. Heldebrant, J. S. Lopez, R. A. Dagle, Frontiers in Energy Research 2023, 11.

[5]	aG. Kresse, J. Furthmüller, Computational Materials Science 1996, 6, 15–50; bG. Kresse, J. Furthmüller, Physical Review B 1996, 54, 11169–11186.

[6]	aJ. P. Perdew, K. Burke, M. Ernzerhof, Physical Review Letters 1996, 77, 3865–3868; bJ. P. Perdew, K. Burke, M. Ernzerhof, Physical Review Letters 1997, 78, 1396–1396.

[7]	S. Grimme, J. Antony, S. Ehrlich, H. Krieg, J Chem Phys 2010, 132, 154104.

[8]	S. L. Dudarev, G. A. Botton, S. Y. Savrasov, C. J. Humphreys, A. P. Sutton, Physical Review B 1998, 57, 1505–1509.

[9]	G. Henkelman, B. P. Uberuaga, H. Jónsson, The Journal of Chemical Physics 2000, 113, 9901–9904.

[10] Wang, V., Xu, N., Liu, J.C., Tang, G., Geng, W.T., VASPKIT: A pre- and post-processing program for VASP code, arXiv:1908.08269.
